# The inflammatory potential of diet in adults with knee osteoarthritis: sex-specific associations with quality of life, sleep, fatigue and mental health

**DOI:** 10.3389/fnut.2025.1624852

**Published:** 2025-09-15

**Authors:** Lynette Law, Joshua J. Heerey, Brooke L. Devlin, Peter Brukner, Alysha M. De Livera, James R. Hebert, Sherry Price, Nathan P. White, Adam G. Culvenor

**Affiliations:** ^1^La Trobe Sport and Exercise Medicine Research Centre, School of Allied Health, Human Services and Sport, La Trobe University, Melbourne, VIC, Australia; ^2^School of Human Movement and Nutrition Sciences, University of Queensland, Brisbane, QLD, Australia; ^3^Department of Mathematics and Statistics, School of Computing, Engineering and Mathematical Sciences, La Trobe University, Melbourne, VIC, Australia; ^4^Cancer Prevention and Control Program, Department of Epidemiology and Biostatistics, Arnold School of Public Health, University of South Carolina, Columbia, SC, United States; ^5^Melbourne Knee Centre, Melbourne, VIC, Australia

**Keywords:** osteoarthritis, chronic inflammation, health-related quality of life, anti-inflammatory diet, dietary inflammatory index, sleep

## Abstract

**Background:**

Knee osteoarthritis (OA) is a disabling condition—characterized by pain, stiffness, and impaired quality of life—that affects more females than males. Chronic systemic inflammation is a key feature of knee OA and can be modulated by diet. We evaluated the sex-specific relationship between the inflammatory potential of diet and health-related quality of life (HRQOL), sleep quality, energy and fatigue levels, and psychological distress in individuals with knee OA.

**Methods:**

This cross-sectional study analyzed baseline data from 144 participants (64% female) aged 45–85 years with symptomatic knee OA enrolled in the FEAST (eFEct of an Anti-inflammatory diet for knee oSTeoarthritis) randomized controlled trial, which compared an anti-inflammatory dietary program to a standard low-fat dietary program. Dietary inflammatory potential was assessed using the dietary inflammatory index (DII^®^) and energy-adjusted DII (E-DII™), calculated from 3-day food diaries. Higher scores reflect more proinflammatory diets. Outcomes were HRQOL (EQ-5D-5L utility score and 100 mm visual analogue scale [VAS]), self-reported sleep quality, energy and fatigue levels (100 mm VAS), and psychological distress (Kessler Scale). Linear regression with interaction terms assessed sex-specific associations between DII/E-DII and outcomes, adjusting for age and body mass index (BMI).

**Results:**

Male and female participants had a mean ± standard deviation BMI of 30.4 ± 3.8 and 30.2 ± 7.2 kg/m^2^, respectively. Mean E-DII and DII were −0.35 ± 1.34 and 0.72 ± 1.49 (females), and −0.26 ± 1.52 and 0.33 ± 1.45 (males). Higher E-DII scores were associated with lower EQ-5D utility scores in females (unadjusted β = −0.03, 95% CI −0.05 to −0.001), but not after adjustment. Higher DII scores were associated with lower energy levels in males (unadjusted β = −4.34, 95% CI −8.44 to −0.23; adjusted β = −4.01, 95% CI −7.91 to −0.30). No associations were found between DII and HRQOL, sleep, fatigue, or psychological distress in either sex.

**Conclusion:**

No associations were found between dietary inflammatory potential and sleep quality, fatigue, or psychological distress. A more proinflammatory diet was linked to lower energy levels in males with symptomatic knee OA. Findings from the FEAST trial will clarify whether reducing dietary inflammation improves these outcomes over time.

## 1 Introduction

Knee osteoarthritis (OA) is a common and disabling condition driven by chronic systemic inflammation (1), leading to joint pain (2), functional limitations (3), and reduced health-related quality of life (HRQOL) (4). It is often accompanied by impaired sleep quality (5), fatigue (6) and depression (7), which exacerbate the overall disease burden. While current treatments focus primarily on symptom management, emerging evidence suggests that dietary interventions may offer modest benefits on physical HRQOL (8). Knee OA outcomes also differ by sex, with females generally reporting worse symptoms and lower HRQOL (9), while males report greater cognitive and mental decline at similar disease stages (10). These disparities may be partly explained by differences in inflammatory responses, as females tend to have higher levels of pro-inflammatory markers and estrogen receptor activity, contributing to increased inflammation and disability (9).

Diet can modulate systemic inflammation by exerting either anti-inflammatory or pro-inflammatory effects (11–13). Anti-inflammatory diets have been linked with greater HRQOL in healthy adults (14), type 2 diabetes (15, 16) and rheumatoid arthritis (17). However, whether similar benefits extend to individuals with knee OA remains unclear. Likewise, while dietary inflammatory potential has been associated with psychological distress (18) and poor sleep quality in healthy adults (19), evidence in chronic conditions, including knee OA, is limited. Exploring these connections in those with knee OA may provide valuable insights into the broader impact of dietary inflammation on wellbeing and inform targeted dietary strategies to enhance quality of life beyond joint health.

The inflammatory potential of diets can be assessed using the dietary inflammatory index (DII^®^) (20) and the more recently developed energy-adjusted DII (E-DII™) (21), which accounts for the confounding effects of total energy intake (20). Our previous work found no associations between diet-related inflammation and knee pain characteristics (22). However, the link between dietary inflammation potential and HRQOL in knee OA remains largely unexplored (8).

Given the high burden of knee OA and its impact beyond the joint, it is critical to understand whether diet-associated inflammation influences these outcomes and if these relationships differ by sex, which may have implications for treatment approaches. This study aimed to assess the sex-specific associations between dietary inflammatory potential and HRQOL, sleep quality, energy and fatigue levels, and psychological distress in adults with symptomatic knee OA.

## 2 Materials and methods

### 2.1 Study design and participants

The current study is an exploratory cross-sectional analysis of baseline data from 144 participants in the FEAST (eFEct of an anti-inflammatory diet for knee oSTeoarthritis) randomized controlled trial, that investigates the effectiveness of an anti-inflammatory dietary program compared to a usual care low-fat dietary program in adults with symptomatic knee OA (8). Eligible participants were aged 45–85 years old with chronic knee pain consistent with a clinical knee OA diagnosis and had no prior total knee arthroplasty in their index knee (i.e., the most symptomatic knee) (23). All participants provided written informed consent prior to participation. This study complies with the Declaration of Helsinki and received approval from La Trobe University Human Research Ethics Committee (HEC-22044).

### 2.2 Measurement of dietary inflammatory potential

Dietary inflammatory potential was assessed using the E-DII and DII. Details regarding the development of E-DII and DII are described elsewhere (21). Briefly, dietary intake data were obtained from self-reported 3-day food diaries collected from participants prior to their baseline FEAST assessment and analyzed using Foodworks^®^ Premium Edition nutrient analysis software (Version 10, Brisbane, Australia 2019). The DII was then calculated for each participant reflecting the inflammatory potential of diet based on 29 (out of a possible 45) food parameters: protein, fat, carbohydrate, alcohol, monounsaturated fatty acids, polyunsaturated fatty acids, selenium, saturated fatty acids, vitamin A, thiamine, riboflavin, niacin, vitamin C, magnesium, iron, zinc, fiber, caffeine, beta carotene, cholesterol, vitamin E, vitamin B12, omega 6, omega-3 fatty acids, trans-fatty acids, folic acid, vitamin B6, energy and vitamin D. When calculated from 25–30 parameters, DII scores range from −5.5 to 5.5 (21), where a higher and positive DII score indicates more proinflammatory diets, and a lower and negative score indicates more anti-inflammatory diets. The DII has been shown to reflect levels of systemic inflammatory markers (e.g., CRP, IL-6, TNF-α) (24). The E-DII was developed to adjust for the influence of total energy intake on dietary inflammatory potential and is calculated per 1,000 kilocalories of energy consumed (21). The E-DII has been validated across multiple studies, showing improved predictive ability for inflammation-related outcomes over the unadjusted DII in various health conditions (21). However, the DII remains widely used in the literature, and including both measures allows for comparisons with previous studies while incorporating the advantages of energy adjustment.

### 2.3 Outcomes

#### 2.3.1 Health-related quality of life

HRQOL was assessed with the EQ-5D-5L (25) utility score and overall rating of health on a visual analogue scale (VAS). The utility score covers five domains: mobility, self-care, usual activities, pain or discomfort, and anxiety or depression. The presence and severity of problems on each domain are rated on a 5-point Likert scale (no problems, slight, moderate, severe, or extreme problems). Responses are used to generate a health profile with 3,125 possible health states defined by combining one level from each dimension, ranging from 11,111 (full health) to 55,555 (worst health) (25). These health states were converted into a single index ‘utility’ score using an Australian value set (26), with possible values ranging from −0.676 (worse than death) to 1.0 (perfect health). The validity and reliability of the EQ-5D has been established in OA populations (27). The EQ-5D VAS asks participants to rate their perceived health today from 0 (the worst imaginable health) to 100 (the best imaginable health) (25).

#### 2.3.2 Assessment of sleep quality, and energy and fatigue levels

Sleep quality, energy, and fatigue levels in the last 7 days were self-reported by participants on a 100-point VAS, each with specific anchors: sleep quality (0 = worst sleep possible, 100 = best sleep possible); energy levels (0 = not at all energetic, 100 = extremely energetic); and fatigue (0 = extremely/always fatigued, 100 = not at all fatigued).

#### 2.3.3 Assessment of participant characteristics

Participant demographic and socioeconomic characteristics, including age, ethnicity, comorbid medical conditions, and employment status were recorded at baseline via self-reported questionnaires. Height and weight were recorded with a stadiometer and column scales, respectively, and used to calculate body mass index [BMI = weight(kg)/height(m)^2^].

#### 2.3.4 Psychological distress

Psychological distress was assessed with the Kessler Psychological Distress Scale (K10) (28), a reliable and valid measure (29). The K10 contains ten items rated on a 5-point Likert scale to yield a global measure of distress and is based on questions concerning anxiety and depression symptoms experienced during the previous four weeks. Total scores range from 10 to 50, with higher scores indicating increased levels of psychological distress. In line with population-based cut-points used by the Australian Bureau of Statistics (30), total scores were grouped into ordered categories of low distress (10–15), moderate distress (16–21), high distress (22–29) and very high distress (30–50).

### 2.4 Statistical analysis

Statistical analysis was conducted using Stata, V.12 (StataCorp, College Station, Texas, USA). Participant characteristics were summarized descriptively. Data were first inspected using plots, such as histograms. Categorical data were presented as percentages with frequency, and continuous data presented using mean and standard deviation. Data found to be skewed were presented using median with IQRs (25th–75th centile). Linear regression models with interaction terms were used to obtain sex-specific associations of the dietary inflammatory indices (E-DII, DII) with EQ-5D (utility score, VAS), sleep quality, energy and fatigue levels, and K-10 score. Models with and without adjustment for covariates were used, and the model coefficients, 95% confidence intervals (CI) and associated *p*-values were reported. In the adjusted models, age and BMI were covariates and chosen *a priori* as they are associated with dietary intake (31, 32) and HRQOL (33).

## 3 Results

Of the 144 participants, 92 (64%) were female, and 52 (36%) were male. Females had mean ± SD age 65 ± 8 years, BMI 30.2 ± 7.2 kg/m^2^, and 80% were classified as overweight/obese (Table 1). Male participants had mean age 67 ± 8 years and BMI 30.4 ± 3.8 kg/m^2^, with the majority (96%) classified as overweight/obese. The mean E-DII was −0.35 ± 1.34 in females and −0.26 ± 1.52 in males, while mean DII was 0.72 ± 1.49 and 0.33 ± 1.45, respectively.

**TABLE 1:** Participant baseline characteristics.

Baseline variable	Females (*n* = 92)	Males (*n* = 52)
Age, years	65 ± 8	67 ± 8
Weight, kg	80.0 ± 19.7	93.8 ± 14.7
Height, cm	163 ± 6	175 ± 8
Body mass index, kg/m^2^	30.2 ± 7.2	30.4 ± 3.8
**Body mass index categories (%)**		
Normal (< 25 kg/m^2^)	18 (20)	2 (4)
Overweight (≥ 25 to < 30 kg/m^2^)	35 (38)	24 (46)
Obese (≥ 30 kg/m^2^)	39 (42)	26 (50)
**EQ-5D**		
Utility score^α^	0.81 ± 0.19	0.85 ± 0.10
VAS^β^	69.2 ± 19.2	71.2 ± 19.0
Self-perceived health indicators*		
Sleep quality	51.8 ± 22.2	61.4 ± 26.3
Energy levels	53.6 ± 22.3	60.3 ± 20.5
Fatigue levels	52.3 ± 23.9	58.7 ± 23.2
E-DII^‡^	−0.35 ± 1.34	−0.26 ± 1.52
DII^∧^	0.72 ± 1.49	0.33 ± 1.45
K10 score^†^	17.1 ± 6.1	14.6 ± 4.1
**K10 scores by category^†^ (%)**		
Low distress (10–15)	45 (49)	35 (67)
Moderate distress (16–21)	27 (30)	13 (25)
High distress (22–29)	16 (17)	3 (6)
Very high distress (30–50)	4 (4)	1 (2)

Values indicate mean (± SD) for continuous variables with normal distribution and *n* (%) for categorical variables. SD, standard deviation; BMI, body mass index; VAS, visual analog scale; DII, dietary inflammatory index, calculated with 29 out of 45 possible parameters; E-DII, energy-adjusted DII; K10, Kessler Psychological Distress Scale.

^α^range −0.676 to 1.0.

^β^Scores range from 0 (the worst health you can imagine) to 100 (the best health you can imagine).

*In the last 7 days, rated on a 100 mm VAS (0 = worst quality, 100 = best quality).

^∧^Range −3.16 to 2.90.

^‡^ Range −2.72 to 3.75.

^†^ In the last month.

Higher E-DII scores were associated with lower energy levels in males (unadjusted β = −3.47, 95% CI −7.41 to 0.46; adjusted β = −4.01, 95% CI −7.91 to −0.30) (Table 2). Higher E-DII scores were also associated with lower EQ-5D utility scores in females (unadjusted β = −0.03, 95% CI −0.05 to −0.001), but not after adjustment (β = −0.02, 95% CI −0.04 to 0.01). DII scores were negatively associated with energy levels in males (unadjusted β = −4.34, 95% CI −8.44 to −0.23), and this association remained after adjustment (β = 0.76, 95% CI −2.12 to 3.64) (Table 3). No associations were found between either the E-DII or DII and EQ-5D VAS, self-reported sleep quality, fatigue levels, or psychological distress (Supplementary Figures 1, 2).

**TABLE 2:** Associations between E-DII, HRQOL, sleep quality, energy and fatigue levels.

	Unadjusted analysis				Adjusted analysis*			
	Male (*n* = 52)		Female (*n* = 92)		Male (*n* = 52)		Female (*n* = 92)	
Variable	β (95% CI)	*p*-value	β (95% CI)	*p*-value	β (95% CI)	*p*-value	β (95% CI)	*p*-value
EQ-5D VAS	0.14 (−3.35 to 3.63)	0.936	−2.04 (−5.00 to 0.92)	0.176	−0.36 (−3.80 to 3.08)	0.836	−1.30 (−4.25 to 1.65)	0.386
EQ-5D-5L utility	0.01 (−0.02 to 0.04)	0.357	−0.03 (−0.05 to −0.001)	**0.045**	0.01 (−0.02 to 0.03)	0.588	−0.02 (−0.04 to 0.01)	0.181
Sleep quality VAS	−2.06 (−6.40 to 2.28)	0.350	0.35 (−3.34 to 4.03)	0.853	−2.33 (−6.71 to 2.05)	0.294	0.74 (−3.02 to 4.49)	0.699
Energy levels VAS	−3.47 (−7.41 to 0.46)	0.083	−0.94 (−4.28 to 2.40)	0.577	−4.01 (−7.91 to −0.30)	**0.034**	0.02 (−3.23 to 3.28)	0.991
Fatigue levels VAS	−0.91 (−5.24 to 3.42)	0.678	−0.95 (−4.62 to 2.72)	0.609	−1.06 (−5.44 to 3.32)	0.633	−0.76 (−4.51 to 3.00)	0.691
Kessler 10	−0.10 (−1.11 to 0.91)	0.846	−0.10 (−0.95 to 0.76)	0.820	−0.11 (−0.85 to 1.07)	0.822	−0.41 (−1.23 to 0.41)	0.327

Linear regression assessed the association between E-DII and baseline characteristics. Bold values indicate *p* < 0.05. CI, Confidence interval; VAS, visual analog scale rated on a 100 mm VAS (0 = worst quality, 100 = best quality).

*Adjusted for age and BMI.

**TABLE 3:** Associations between DII and HRQOL, sleep quality, energy and fatigue levels.

	Unadjusted analysis				Adjusted analysis*			
	Male (*n* = 52)		Female (*n* = 92)		Male (*n* = 52)		Female (*n* = 92)	
Variable	β (95% CI)	*p*-value	β (95% CI)	*p*-value	β (95% CI)	*p*-value	β (95% CI)	*p*-value
EQ-5D VAS	−0.67 (−4.35 to 3.00)	0.719	−0.01 (−0.03 to 0.02)	0.837	−1.44 (−5.06 to 2.19)	0.435	0.75 (−1.88 to 3.37)	0.576
EQ-5D-5L utility score	−0.02 (−0.02 to 0.05)	0.338	−0.01 (−0.03 to 0.02)	0.719	0.01 (−0.02 to 0.04)	0.537	0.01 (−0.02 to 0.03)	0.642
Sleep quality VAS	−2.04 (−6.58 to 2.50)	0.376	−1.09 (−4.20 to 2.21)	0.514	−2.44 (−7.05 to 2.17)	0.297	−0.94 (−4.29 to 2.40)	0.577
Energy levels VAS	−4.34 (−8.44 to −0.23)	**0.039**	0.007 (−2.98 to 2.99)	0.996	−5.10 (−9.08 to −1.13)	**0.012**	0.76 (−2.12 to 3.64)	0.603
Fatigue levels VAS	−2.57 (−7.07 to 1.93)	0.261	−1.55 (−4.82 to 1.73)	0.352	−2.93 (−7.51 to 1.64)	0.207	−1.55 (−4.87 to 1.78)	0.357
Kessler 10	0.12 (−0.92 to 1.17)	0.819	0.46 (−0.30 to 1.22)	0.236	0.38 (−0.63 to 1.40)	0.453	0.27 (−0.46 to 1.01)	0.459

Linear regression assessed the association between DII and baseline characteristics. Bold values indicate *p* < 0.05. CI, Confidence interval; VAS, visual analog scale rated on a 100 mm VAS (0 = worst quality, 100 = best quality).

*Adjusted for age and BMI.

## 4 Discussion

This cross-sectional study is the first to explore the association between dietary inflammatory potential and key quality of life outcomes in males and females with symptomatic knee OA. Higher E-DII scores (reflecting more pro-inflammatory diets) were associated with lower energy levels in males. Similarly, higher DII scores were associated with lower energy levels in males, but given the paucity of previous research in this area, the clinical significance of these findings are unknown. No associations were found between DII and HRQOL, sleep quality, fatigue, or psychological distress in either sex. These findings are partly consistent with our previous work (22), which found no evidence of an association between dietary inflammatory potential and knee-specific outcomes, medical comorbidities, and symptomatic musculoskeletal sites (22). Notably, the inverse association between E-DII and EQ-5D utility scores observed in females was attenuated after adjusting for age and BMI. This highlights the importance of considering demographic and anthropometric factors when interpreting diet-health relationships and may partly explain the lack of consistent sex-specific findings.

There is limited evidence in knee OA populations for direct comparison with our findings. A study by Toopchizadeh et al. (34) conducted in a slightly larger cohort of knee OA patients (*n* = 220) reported an association between higher DII tertiles and lower HRQOL as assessed by the SF-36 instrument. A cross-sectional analysis conducted in a healthy population found that a pro-inflammatory diet (mean E-DII −1.28 ± 1.51) was associated with greater risk of worse mental health outcomes, including anxiety, psychological wellbeing and depressive symptoms (18), particularly in females, whereas no significant associations were found in males (18). The overall diets in that study were more inflammatory than the FEAST cohort, which had a mean E-DII score −0.31 ± 1.40. Our recent meta-analysis concluded that anti-inflammatory dietary interventions result in small improvements in physical component scores of HRQOL but not mental component scores of HRQOL (8). While we used the Kessler instrument to assess psychological distress, our current study aligns in part with our review as we found no relationship between dietary inflammatory potential and mental health.

While chronic systemic inflammation is strongly tied to sleep disturbances (35, 36), evidence specifically linking dietary inflammation to sleep remains inconsistent (37). Higher DII scores have been linked with poorer sleep in females with overweight and obesity (38), while other studies conducted in healthy adults (39–41) and individuals with sleep apnoea (42) reported no significant association between DII scores and sleep. A larger cohort study reported links between higher DII and poorer sleep quality, particularly in individuals with overweight or obesity, a group characterized by chronic low-grade inflammation (43). Several factors may explain the lack of associations observed in this study. Sleep is regulated by complex physiological mechanisms, with inflammation being only one of many contributing factors. Pain and depressive symptoms—both highly prevalent in those living with knee OA—may have a more pronounced impact on sleep quality than dietary inflammation (5, 7). Additionally, the use of self-reported sleep and dietary intake data introduces the potential for measurement bias (44). Importantly, while the E-DII is a validated tool for estimating dietary inflammatory potential, it may not capture other aspects of dietary behavior that could influence sleep, energy, and fatigue, such as meal timing (45) and specific nutrient profiles (46). The narrow range of DII and E-DII scores observed in the FEAST cohort may also have limited our ability to detect associations, particularly when compared with studies that included individuals with more proinflammatory diets. Furthermore, other factors such as physical limitations (47) and mental health status (48) appear to be strongly linked to fatigue rather than systemic inflammation alone in knee OA. This supports the view that fatigue is a multifactorial construct that may not be fully captured by single-item scales like a VAS (49).

In addition to improved disease-specific outcomes, a reported benefit of lowering dietary inflammatory potential is improved energy levels and less fatigue (50). We found that higher dietary inflammatory potential was linked to lower energy levels in male participants, but no associations with fatigue levels in either sex. This sex-specific association between a more proinflammatory diet and reduced energy levels in males is consistent with previous research conducted in healthy Australian adults, which reported associations between diet quality (assessed using the dietary guideline index and recommended food score) and energy levels in men (51). While these indices reflect overall diet quality rather than dietary inflammatory potential specifically, the overlap suggests that proinflammatory diets may lack key nutrients involved in brain function and energy metabolism, such as omega-3 fatty acids and B vitamins (52). While this suggests that anti-inflammatory dietary patterns may play a role in supporting energy levels in men with knee OA, no studies to date have established whether such changes in self-reported energy levels (as linked to dietary inflammatory potential) translate to meaningful impacts on function or quality of life. Therefore, the clinical significance of this association remains unclear. Further research is needed to confirm these sex-specific effects and understand the underlying mechanisms.

Despite well-documented sex differences in knee OA severity and symptoms (9, 53), we found few differences in the relationships with dietary inflammatory potential in males and females. Studies consistently show that females exhibit higher levels of proinflammatory markers (e.g., interleukin-6 and tumor necrosis factor-α) compared to males (54). It is possible that the effects of diet-related inflammation are overshadowed by other dominant drivers of sex differences in knee OA outcomes. For example, hormonal changes—particularly post-menopausal declines in estrogen—have been linked to knee OA incidence, pain sensitivity and inflammatory responses in females (9), partly due to estrogen’s regulatory role in immune modulation and cytokine expression. Males and females also differ in inflammatory metabolism, with females exhibiting higher circulating levels of proinflammatory cytokines, such as IL-6 and TNF-α (54). These sex-specific immune and hormonal differences may influence the body’s response to dietary inflammation, potentially explaining why associations with energy levels were observed in males but not females. Sex differences in body composition and central pain processing could also contribute to variations in knee OA severity (9).

Our results should be interpreted with caution given the exploratory nature of this analysis from baseline FEAST trial data. Limitations include the potential for our analyses to be underpowered, as the sample size calculation was based on the main trial outcome (12-week change in knee symptoms) and may lead to insufficient statistical power for the sex-specific associations. While the EQ-5D and Kessler 10 are valid tools for assessing HRQOL and psychological distress, respectively, we used a single VAS question to assess sleep quality and levels of energy and fatigue. While validated instruments for these measures exist [e.g., the Pittsburgh Sleep Quality Index (55), Multidimensional Fatigue Inventory (56)], we were constrained to a more simple measure to minimize participant burden in the larger trial. The use of validated multi-item instruments (e.g., the Pittsburgh Sleep Quality Index or Multidimensional Fatigue Inventory) in future research may improve measurement precision and construct validity. It is well-established that self-report diet data is notoriously difficult to validate (57), but all 3-day food diary data in the FEAST trial was diligently checked and clarified with participants to minimize misreporting and missing data. We were also only able to include 28 and 29 of the total 45 parameters that make up the E-DII and DII score, respectively, due to constraints of the nutrient composition databases in Foodworks, which did not contain data for several DII components. Many of the missing parameters—such as turmeric and flavonoids–were not available, limiting our ability to compute the full DII/E-DII. However, research shows that there is no change in predictive ability when at least 25 parameters are used (58). The 16 missing components are also all anti-inflammatory, and some may have a beneficial effect on knee OA symptoms, which may influence the associations observed (59, 60). In light of these shortcomings, it is important to note that the ranges of DII and E-DII scores (−2.72 to 3.75 and −3.16 to 2.90, respectively) are very narrow compared to other studies (18, 61). It is known in epidemiologic studies that a very narrow range of exposure will bias the results toward the null (62, 63). Lastly, while BMI was used as a measure of adiposity in this study, we acknowledge that assessing body composition, particularly fat mass and fat-free mass, could have provided more precise insights into the link between diet, systemic inflammation, and knee osteoarthritis. Future studies incorporating detailed body composition measures may help clarify these relationships further.

## 5 Conclusion

Dietary inflammatory potential, measured by the E-DII and DII, was generally not associated with HRQOL, self-perceived health indicators of sleep, fatigue, or psychological distress in males or females with symptomatic knee OA. A higher proinflammatory diet score was associated with lower energy levels in males but given the lack of an established minimum clinically important difference (MCID) for single-item energy VAS measures in knee OA populations, the clinical significance of this association is currently uncertain. The results of the FEAST trial will reveal if reducing inflammatory potential of diet improves these important health outcomes longitudinally.

## Data Availability

The raw data supporting the conclusions of this article will be made available by the authors, without undue reservation.
