# Supplementary material for: The inflammatory potential of diet in adults with knee osteoarthritis: sex-specific associations with quality of life, sleep, fatigue and mental health
Source: Front Nutr. 2025 Sep 15;12:1624852. doi: 10.3389/fnut.2025.1624852 (PMC12476995; doi:10.3389/fnut.2025.1624852)

Supplementary Figure 1. Scatterplots describing E-DII associations by sex

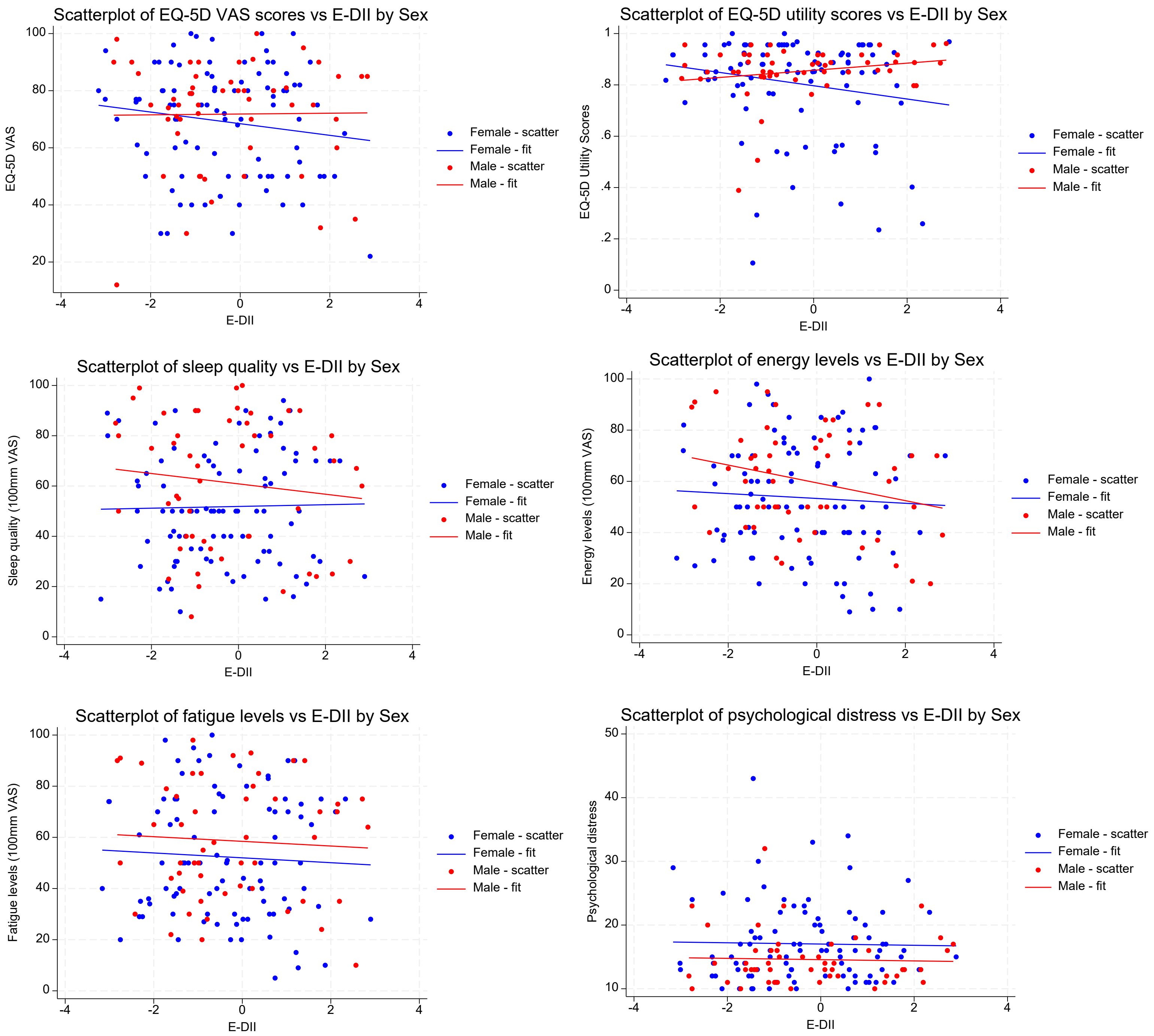

Supplementary Figure 2. Scatterplots describing DII associations by sex

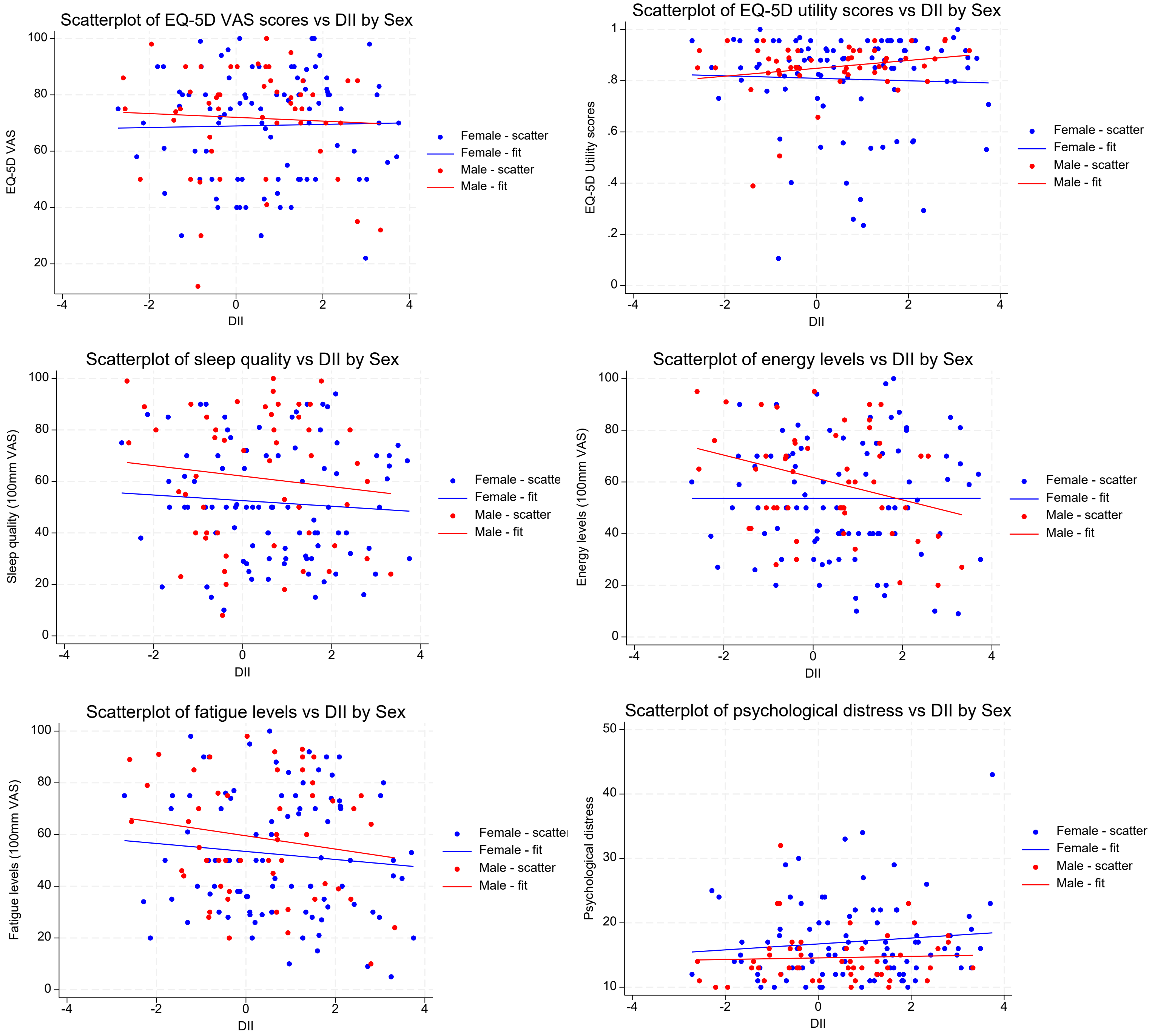

Supplement: Supplementary file 1 [file Image_1.pdf]
